# Cell Cycle Machinery in *Bacillus subtilis*

**DOI:** 10.1007/978-3-319-53047-5_3

**Published:** 2017-05-13

**Authors:** Jeff Errington, Ling Juan Wu

**Affiliations:** Centre for Bacterial Cell Biology, Institute for Cell and Molecular Biosciences, Faculty of Medical Sciences, Newcastle University, Newcastle upon Tyne, NE2 4AX UK

**Keywords:** Bacillus, *B. subtilis*, MreB, MreB homologues, Bacterial cell shape, Helical filaments, Circumferential motion, Cell elongation machinery, Peptidoglycan synthesis, PG, Divisome, Min system, MinJ, FtsZ, Z ring, Sporulation, SpoIIE, L-form bacteria

## Abstract

*Bacillus subtilis* is the best described member of the Gram positive bacteria. It is a typical rod shaped bacterium and grows by elongation in its long axis, before dividing at mid cell to generate two similar daughter cells. *B. subtilis* is a particularly interesting model for cell cycle studies because it also carries out a modified, asymmetrical division during endospore formation, which can be simply induced by starvation. Cell growth occurs strictly by elongation of the rod, which maintains a constant diameter at all growth rates. This process involves expansion of the cell wall, requiring intercalation of new peptidoglycan and teichoic acid material, as well as controlled hydrolysis of existing wall material. Actin-like MreB proteins are the key spatial regulators that orchestrate the plethora of enzymes needed for cell elongation, many of which are thought to assemble into functional complexes called elongasomes. Cell division requires a switch in the orientation of cell wall synthesis and is organised by a tubulin-like protein FtsZ. FtsZ forms a ring-like structure at the site of impending division, which is specified by a range of mainly negative regulators. There it recruits a set of dedicated division proteins to form a structure called the divisome, which brings about the process of division. During sporulation, both the positioning and fine structure of the division septum are altered, and again, several dedicated proteins that contribute specifically to this process have been identified. This chapter summarises our current understanding of elongation and division in *B. subtilis*, with particular emphasis on the cytoskeletal proteins MreB and FtsZ, and highlights where the major gaps in our understanding remain.

## Introduction to *B. subtilis*

*Bacillus subtilis* is an aerobic, Gram positive, endospore forming bacterium of the phylum Firmicutes. It is by far the best characterised Gram positive organism and basic knowledge about *B. subtilis* is frequently used to guide and inform thinking about other Gram positive organisms. Historically, interest in *B. subtilis* was based largely on three features of its biology: early success in achieving natural transformation with linear DNA, which greatly facilitated genetic analysis of the organism (Anagnostopoulos and Spizizen 1961); its ability to form endospores, which was used as a simple model for cellular development and differentiation (Errington 1993, 2003; Tan and ramamurthi 2014); and industrial interest in its prodigious ability to secrete certain valuable hydrolytic enzymes (e.g. proteases and amylases) directly into the growth medium (Pohl and Harwood 2010).

**Fig. 3.1:**
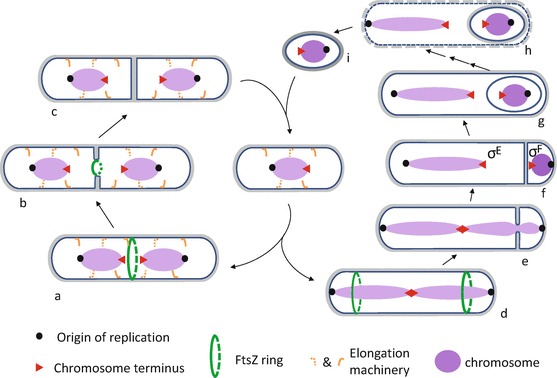
*B. subtilis* cell cycle. The *left half* represents the vegetative cycle, where a new born cell (*centre*) grows in length, controlled by the elongation machinery (*orange curved lines*), in the meantime the chromosome is replicated and FtsZ (*green ring*) assembles between segregated chromosomes at mid cell (*a*). As cell division progresses and septum grows inwards, the Z ring contracts (*b*). Upon completion of septation, which generates two identical daughter cells, the Z ring and the divisome disassemble (*c*) and the dividing wall splits to allow separation of the new born cells (*centre*). Under starvation conditions *B. subtilis* cells initiate spore development (*right half*). Instead of segregating the replicated chromosomes to quarter positions, the sister chromosomes undergo a conformational change to form an elongated structure called the axial filament, which extends from pole to pole. FtsZ assembles at two sub-polar positions, one at each cell pole (*d*). Only one of the two Z rings develops into a septum, which forms over one of the chromosomes (*e*). Following the completion of asymmetric septation, which generates two unequal sized daughter cells, the small prespore or forespore and the large mother cell, transcription factor Sigma F (σ^F^) is activated in the prespore and the remaining part of the prespore chromosome is segregated. Activation of σ^F^ in the prespore leads to the activation of Sigma E ( σ^E^) in the mother cell (*f*). The different programmes of gene expression in the prespore and the mother cell direct the engulfment of the prespore by the mother cell (*g*). Finally, the spore undergoes maturation, and the mother cell lyses (*h*) to release a highly resistant, dormant spore (*i*), which can germinate and start growing (*centre*) when nutrients become available

The biggest driver for study of *B. subtilis*, at least in the 1960s to 1990s, was probably interest in endospore (spore) formation (Fig. 3.1). Sporulation of *B. subtilis* is triggered essentially by nutrient stress. The process begins with a modified, highly asymmetric cell division. This generates a small prespore (sometimes called forespore) cell, destined to become the mature endospore, and a much larger mother cell. The mother cell engulfs the prespore, forming a cell within a cell. The two cells then cooperate in a complex developmental process in which the prespore becomes highly differentiated and covered in protective layers. Eventually, the mother cell lyses to release the now dormant endospore. Endospores are incredibly resistant and can remain dormant for extremely long periods of time, before germinating in response to specific chemical signal molecules (germinants). The process of sporulation in *B. subtilis* is now understood in great detail (Errington 1993, 2003; Tan and Ramamurthi 2014).

Research on spore formation contributed considerably to the development of methods for studying the sub-cellular distribution of proteins and other important macromolecules in bacteria, laying the foundations for modern bacterial cell biology (Shapiro and Losick 2000; Errington 2003). These imaging methods, together with the exceptionally powerful molecular genetics of *B. subtilis*, stimulated a new era of studies on the cell cycle and cell morphogenesis. FtsZ, a tubulin homologue that is the key player in bacterial cell division, and MreB, an actin homologue that governs cell shape in many rod shaped bacteria, will be the main topics for discussion in this chapter. As the main focus of the chapter lies on *B. subtilis*, reference to work on other bacteria will be limited to situations where the contrast or additional information is helpful. For more detail on the *E. coli* system and on FtsZ and MreB proteins generally, the reader is directed to Chaps. 10.1007/978-3-319-53047-5_2, 10.1007/978-3-319-53047-5_5, 10.1007/978-3-319-53047-5_7 and 10.1007/978-3-319-53047-5_8.

## MreB and the Cell Elongation Machinery

### Organization of the *B. subtilis* Cell Wall

Peptidoglycan (PG) is the major component of virtually all bacteria (Typas et al. 2012). It comprises a single huge macromolecule that covers the entire surface of the cell. Lying just outside the cytoplasmic membrane it acts as a protective layer but it also constrains the membrane against the outward turgor pressure imposed by the high osmolarity of the cytoplasm. PG is of considerable practical significance as its synthesis is the target for many useful antibiotics, and fragments of the wall are recognised by innate immune receptors during infection. The PG contributes to the shape of the cell but has no intrinsic 3D shape, so it must be sculpted by synthetic enzymes into the correct form.

PG is composed of long glycan strands with alternating N-acetylglucosamine and N-acetylmuramic acid sugars, cross linked by peptide bridges made up of a mixture of L- and D-amino acids (De Pedro and Cava 2015). The precursor for PG synthesis, called lipid II, is a disaccharide pentapeptide coupled to a C_55_ isoprenoid lipid (bactoprenol) and is synthesised in the cytosol by a well characterised series of enzymes. Lipid II is flipped to the exterior and assembled into the existing cell wall sacculus by a multiplicity of synthetic enzymes called penicillin-binding proteins (PBPs), which possess the glycosyltransferase and transpeptidase activities needed to extend the glycan strands and create peptide cross bridges (Lovering et al. 2012; Scheffers and Tol 2015). Recently the RodA protein was identified as a possible monofunctional glycosyltransferase (Meeske et al. 2016 and Emami et al. 2017). Extracellular autolytic enzymes are required to allow expansion of the wall by breaking bonds in pre-existing material. Their activities need to be tightly regulated to enable controlled expansion of the wall during growth, while avoiding potentially catastrophic turgor-driven lysis (Vollmer et al. 2008).

Gram positive bacteria lack the outer membrane characteristic of Gram negatives. However, Gram positive walls typically contain a second major class of polymers called teichoic acids (TAs) (Sewell and Brown 2014; Percy and Grundling 2014). In many Gram positives there are two major forms: wall teichoic acids (WTA), which are covalently linked to the PG; and lipoteichoic acids (LTA), which are coupled to a lipid carrier. In *B. subtilis* WTA and LTA have the same general composition, of poly-[glycerol-phosphate]. TAs have been implicated in many functions. Metal homeostasis is probably a central role – scavenging divalent cations and maintaining surface charge.

*B. subtilis* cells have a characteristic morphology; in essence, an elongated regular cylindrical tube with hemispherical poles. Growth occurs by elongation along the long axis of the cell, and division occurs approximately each time a doubling in length occurs. The cells have a typical diameter of about 850 nm, irrespective of the growth rate. Changes in growth rate are accommodated by alterations in cell length, with faster growing cells (average up to about 5 μm) being longer than slower growing cells (average minimally about 2 μm) (Sharpe et al. 1998).

Cell shape determination is a central problem in biology. In *B. subtilis* and many other rod-shaped bacteria, shape is thought to be determined and maintained by the action of “cytoskeletal” proteins of the MreB family (see below). These proteins are structurally and biochemically related to eukaryotic actins. Like actin proteins, they undergo reversible polymerization, which is regulated by binding and hydrolysis of ATP. However, recent work has highlighted certain intrinsic differences, most notably the tight direct association of MreB polymers with the cytoplasmic membrane (Salje et al. 2011). Mutations in the *mreB* genes of many bacteria abolish proper cell shape control. The possibility that they have a direct role in cell shape determination and or maintenance comes in part from the analogy with shape control by actin filaments in eukaryotes but also from the view that the organization of a large scale (μm) structure requires long range topographical instructions, such as might be provided by elongated MreB filaments. Early experiments examining the localization of MreB proteins in *B. subtilis* provided strong support for this view through the observation of elongated helical filaments that appeared to wrap around the long axis of the cell (Jones et al. 2001). However, the significance of these elongated filaments and even their existence have been the subject of much recent debate (see below).

### *B. subtilis* Has Three Actin Like MreB Homologues

The *mreB* gene was first defined by mutations altering cell shape in *E. coli* (Wachi et al. 1987). Early genome sequence analyses revealed that *B. subtilis* has three *mreB* paralogous genes (Levin et al. 1992; Varley and Stewart 1992; Abhayawardhane and Stewart 1995). The gene designated *mreB* has an equivalent chromosomal location to that of *mreB* genes of most other bacteria, in lying immediately upstream of homologues of *mreC* and *mreD* genes that are also involved in cell elongation. The other homologues – *mbl* (MreB like) and *mreBH* (MreB homologue) are located in distant parts of the chromosome. Early mutational studies of *mreB* and *mbl* were hampered by the presence of the important *mreC* and *mreD* genes downstream from *mreB* and the lethal nature of the mutations. The latter problem was simplified by the finding that for both paralogues, the viability of null mutants can be rescued by addition of high concentrations (e.g. 20 mM) of Mg^2+^ to the culture medium, for reasons that are not clear (Formstone and Errington 2005). To summarise the results of several papers, the three paralogues appear to have partially redundant functions, and overexpression of any one of the genes can enable growth and reasonably normal morphology of an otherwise triple null mutant. Single mutations tend to have subtly different effects on morphology: *mreB* mutants have an increased diameter but remain able to grow in a straight line; *mbl* mutants are highly twisted with some bulging and lysis; *mreBH* mutants have a narrow cell phenotype, especially under low Mg^2+^ conditions (Jones et al. 2001; Carballido-López et al. 2006; Kawai et al. 2009a; Defeu Soufo and Graumann 2006).

Interestingly, Schirner and Errington (2009) found that upregulation of an ECF sigma factor (σ^I^) involved in cell envelope stress was sufficient to suppress the lethality that normally occurs in attempting to construct an *mreB*, *mbl*, *mreBH* triple mutant. The suppressed mutant grew well (though requiring high Mg^2+^) but with a spherical morphology. Thus, it seems that, as in many other organisms, MreB proteins play a crucial role in rod-shape morphogenesis and cylindrical cell wall extension.

### Filaments, Foci and Movement

Early imaging experiments with all three *B. subtilis* MreB family proteins appeared to reveal extended helical filaments localized close to the cell periphery and thus presumably close to the inner surface of the cytoplasmic membrane (Jones et al. 2001). All three MreB family proteins exhibited roughly similar patterns of localization and seemed to co-localize, at least under some conditions (Carballido-López et al. 2006; Defeu Soufo and Graumann 2006). The localization of various cell elongation proteins (Leaver and Errington 2005; Formstone et al. 2008) and the use of labelling methods designed to identify nascent cell wall synthesis (Daniel and Errington 2003; Tiyanont et al. 2006) also seemed compatible with a helical mode of wall synthesis. Some experiments suggested a degree of remodelling or active movement of the filaments during cell elongation (Carballido-López and Errington 2003; Defeu Soufo and Graumann 2004). The filamentous helical view of MreB localization was exciting because, in principle, the filaments could act as a geometric guide for the synthetic machinery directly defining the morphology of rod-shaped cells.

In 2011, however, three groups described experiments that revealed the circumferential movement of relatively short filaments or foci (rather than long filaments) of MreB proteins in *B. subtilis* (Garner et al. 2011; Domínguez-Escobar et al. 2011) and *E. coli* (Van Teeffelen and Gitai 2011). Importantly, the circumferential movement was dependent on active cell wall synthesis, suggesting that MreB follows rather than leads the synthetic process. The problem with the short filament or patchy view of MreB organization lies in the question of what its role is in cell elongation. Domínguez-Escobar et al. (2011) proposed that MreB acts as a scaffold to help assemble the complex of proteins needed to coordinate the synthesis of PG, WTA and other wall associated elements. The complex was proposed to use existing glycan strands as a template for insertion of new material. However, this presumably only works while the template strands have the correct geometry and could not account for the long term fidelity of shape maintenance and especially the restoration of shape in cells with any shape abnormality.

Meanwhile, electron cryotomography failed to detect elongated filaments of native MreB in flash frozen intact *E. coli* cells (Swulius et al. 2011), and demonstrated that the very prominent helical cytoplasmic filaments made by one particular *E. coli* MreB/GFP fusion protein were an artefact specific to that fusion protein (Swulius and Jensen 2012). However, Salje et al. (2011) potentially solved the conundrum of the missing filaments of native protein in the cryo-EM experiments through the discovery that MreB protein polymers have a high affinity for membranes and in vivo this tight membrane association would likely hide the filaments from detection in the low contrast cryo-EM images. This also explained the problem with the *E. coli* GFP-MreB fusion protein mentioned above because the membrane targeting sequence of *E. coli* MreB is close to the N-terminus and thus probably occluded by the GFP fusion. Meanwhile, Dempwolff et al. (2011) obtained supporting evidence for membrane association of MreB and Mbl, though interestingly not MreBH, by expressing GFP fusions of the 3 proteins in eukaryotic cells.

The most recent in vivo imaging experiments on *B. subtilis*, using various super-resolution methods may have resolved some of the confusion around MreB localization, by demonstrating that the proteins (at least MreB and Mbl) are able to form relatively extended helical filaments, at least under some conditions, but that the whole filament systems undergo overall near circumferential movement, which is dependent on (and presumably driven by) PG synthesis (Reimold et al. 2013; Olshausen et al. 2013). Olshausen et al. (2013) suggested that the elongated filaments could serve to coordinate the synthetic activities of multiple wall synthetic complexes, providing a plausible mechanistic explanation for the function of long MreB filaments.

Two recent lines of work on the *E. coli* MreB system suggest that MreB may have more than one mode of action in cell shape regulation, which may clarify some of the conflicting data described previously. First, Ursell et al. (2014) and Billings et al. (2014) found that MreB filaments or patches have affinity for regions of a particular (aberrant) curvature. Recruitment of the cell wall machinery to those sites could help to correct the curvature leading to the restoration of shape. Morgenstein et al. (2015) then discovered that in certain mutational backgrounds, MreB motion is not required for maintenance of a rod shape. The authors proposed that MreB can help specify cell shape by two distinct mechanisms: first, by a motion-independent mechanism that relies on recruitment of the synthetic machinery to sites of inappropriate curvature, effectively a “repair” mechanism; and second, by a motion-dependent mechanism that helps distribute synthesis over a greater proportion of the surface and is used when cells are growing rapidly and presumably are relatively unperturbed.

The significance of movement and the possible roles of filaments in shape determination have been reviewed in detail recently (Errington 2015).

### A Complex Web of Interactions Between MreB Proteins and Cell Wall Effectors

The models discussed above mainly assume that MreB proteins work by controlling the spatial activity of the enzymes responsible for cell wall expansion. This view is supported by numerous reports describing interactions between MreB and various components of the cell wall machinery. The list of possible MreB interacting proteins identified by various methods as candidate members of the *B. subtilis* elongation machine or “elongasome” are summarised in Table 3.1. Similar collections of interacting proteins have been identified for several other rod-shaped organisms, particularly *E. coli* and *Caulobacter crescentus* (reviewed by (Errington 2015). The methods used to identify these proteins include various indirect approaches, such as co-localization, localization dependence, and genetic epistasis, as well as more direct methods of bacterial or yeast 2-hybrid approaches and biochemical pull downs. Although some of the data are not entirely convincing, for example, the “helix-like” localization patterns, taken together, these methods point to the existence of large elongasome complexes containing multiple proteins involved in synthesis of PG and WTA, as well as potentially extracellular factors, such as autolytic enzymes or their regulators. At the moment, little is known about the stoichiometry of these complexes or about whether they are stable or transient.

**Table 3.1:** Possible MreB interacting poteins identified as candidate components of the elongasome

Protein^a^	MW (kDa)	Localization^b^	Comment	References
MreC	32	I/E	Cell shape function. Encoded by gene immediately downstream from *mreB*	Leaver and Errington (2005), Kawai et al. (2009b), Garner et al. (2011) and Domínguez-Escobar et al. (2011)
MreD	19	I	Cell shape function. Encoded by gene immediately downstream from *mreBC*	Leaver and Errington (2005), Garner et al. (2011), Domínguez-Escobar et al. (2011) and Muchova et al. (2013)
RodZ	23	I/C	Required for normal cell shape	Domínguez-Escobar et al. (2011) and Muchova et al. (2013)
CwlO	50	E	Autolytic enzyme, regulated by FtsEX	Domínguez-Cuevas et al. (2013)
LytE	37	E	Autolytic enzyme. Export regulated by MreBH?	Carballido-López et al. (2006)
FtsE	25	C	ABC-transporter (ATP-binding protein). With FtsX regulates CwlO. Controlled specifically by Mbl?	Domínguez-Cuevas et al. (2013)
FtsX	32	I	ABC-transporter (membrane protein). With FtsE regulates CwlO. Controlled specifically by Mbl?	Domínguez-Cuevas et al. (2013)
PBP 1	99	E	Major bifunctional PBP. Important for both cell elongation and division	Van Den Ent et al. (2006) and Kawai et al. (2009a, b)
PBP 2A	79	E	Major TPase with specific role in elongation. Partially redundant to PBP H	Van Den Ent et al. (2006), Kawai et al. (2009a, b), Garner et al. (2011) and Domínguez-Escobar et al. (2011)
PBP 2B	79	E	Major TPase with specific role in division	Van Den Ent et al. (2006) and Kawai et al. (2009b)
PBP 2C	79	E	Bifunctional PBP with unknown function	Van Den Ent et al. (2006) and Kawai et al. (2009b)
PBP 2D	71	E	Transpeptidase with unknown function	Van Den Ent et al. (2006) and Kawai et al. (2009b)
PBP 3	74	E	Accessory TPase that can rescue cell division in the absence of PBP 2B activity	Kawai et al. (2009b)
PBP 4	70	E	Bifunctional PBP with unknown function	Kawai et al. (2009a, b)
PBP H	76	E	Major TPase with specific role in elongation. Partially redundant to PBP 2A	Van Den Ent et al. (2006), Kawai et al. (2009b), Domínguez-Escobar et al. (2011)
PBP I	65	E	TPase of unknown function.	Van Den Ent et al. (2006) and Kawai et al. (2009b)
RodA	43	I	PG synthesis. Possible monofunctional GTase	Domínguez-Escobar et al. (2011), Meeske et al. (2016), Emami et al. (2017)
DapI	41	C	N-acetyl-diaminopimelate deacetylase. PG synthesis	Rueff et al. (2014)
TagA	29	C	Teichoic acid synthesis. UDP-N-acetyl-D-mannosamine transferase	Formstone et al. (2008)
TagB	44	C	Teichoic acid synthesis. Putative CDP-glycerol:glycerol phosphate glycerophosphotransferase	Formstone et al. (2008)
TagF	87	C	Teichoic acid synthesis. CDP-glycerol:polyglycerol phosphate glycero-phosphotransferase	Formstone et al. (2008)
TagG	32	I	ABC transporter for teichoic acid translocation (permease)	Formstone et al. (2008)
TagH	59	C	ABC transporter for teichoic acid translocation (ATP-binding protein)	Formstone et al. (2008)
TagO	39	C	Teichoic acid synthesis. Undecaprenyl-phosphate-GlcNAc-1-phosphate transferase	Formstone et al. (2008)
TagT	35	E	Transfer of anionic cell wall polymers from lipid-linked precursors to peptidoglycan	Kawai et al. (2011)
TagU	34	E	Transfer of anionic cell wall polymers from lipid-linked precursors to peptidoglycan	Kawai et al. (2011)
YvcK	34	C	Required for normal localization of PBP 1	Foulquier et al. (2011)
GpsB	11	C	Regulation of PBP 1 localization, especially its switch between elongation and division sites.	Claessen et al. (2008)
EF-Tu	43	C	Translation elongation factor	Defeu Soufo et al. (2015)

^a^In addition to the above, Kawai et al. (2011) identified many additional MreB-associated proteins by pull-down mass spectrometry

^b^ *I* integral membrane, *E* extracellular, *C* cytoplasmic

### The Future

Much remains to be learned about the detailed functions of the MreB family proteins of *B. subtilis*. It is by no means clear why *B. subtilis* possesses three paralogous genes. At one level, it reflects the general complexity of the cell wall synthetic machinery of the organism. Thus, it also carries multiple copies of many other synthetic genes, including, for example: 4 class A (TPase and GTase) PBPs (Popham and Setlow 1996), 3 LTA synthases (Grundling and Schneewind 2007a, b; Schirner et al. 2009), 3 WTA transferases (Kawai et al. 2011), and at least 2 families of lipid II flippases (Meeske et al. 2015). The overlapping semi-redundant functions of the 3 MreB proteins may reflect that they interact differentially with subsets of cell envelope proteins in order to adapt cell envelope properties to changing environmental conditions. Perhaps “chemical warfare” between organisms in complex and highly competitive environments such as soil, drives adaptability in cell envelope synthesis and organization. Although some aspects of the differential activities of the 3 MreB proteins are beginning to be worked out (Carballido-López et al. 2006; Domínguez-Cuevas et al. 2013), much more probably remains to be elucidated.

An important related question concerns how the many interactions between MreB proteins and the various other components of the cell envelop synthetic machinery (PG synthases, PBPs, autolysins, WTA synthases, *etc*) are mediated, particularly whether they are static or dynamic and the extent to which they are hierarchical and mutually permissive or exclusive.

A final major question concerns the localization and dynamic properties of the proteins. What conditions determine the length of the MreB filaments and how do length and movement relate to the various problems associated with cell shape determination, maintenance and repair?

There is a sense that the array of analytical methods we now possess, enabling us to localise proteins with increasing temporal and spatial resolution and to define the components of protein complexes and their stoichiometry, should allow details of the machinery and mechanisms to be resolved. Complexity may be the biggest barrier to progress.

## FtsZ and the Cell Division Machinery

Most bacteria with a PG wall divide by directing the ingrowth of a sheet of wall material that eventually forms the new hemispherical poles of the daughter cells. In almost all bacteria, the key cytoskeletal protein involved in defining the site of division and then orchestrating the process is called FtsZ, which is structurally and biochemically homologous to tubulin (Löwe and Amos 1998). In bacteria where the process has been studied in detail, FtsZ appears to form a circumferential ring that defines the site of cell division (Bi and Lutkenhaus 1991). It also serves to recruit, directly or indirectly, multiple protein components of a division machine, sometimes called the “divisome” (Adams and Errington 2009; Egan and Vollmer 2013). Several divisome associated proteins might also be considered as cytoskeletal proteins (e.g. FtsA, DivIVA, MinD; see below), depending on the definition. *B. subtilis* is an interesting model for the study of bacterial cell division because it has two contrasting modes of division: a “conventional mode”, carried out by vegetatively growing cells; and a modified, highly asymmetric division undertaken by sporulating cells.

### Biochemical Properties of FtsZ

The presence of a tubulin GTP-binding signature motif in FtsZ (GGGTGTG) was first reported in the early 1990s (Raychaudhuri and Park 1992; De Boer et al. 1992; Mukherjee and Lutkenhaus 1994). Crystallographic studies confirmed the near congruence of the structures of FtsZ and tubulin proteins (Löwe and Amos 1998). Not surprisingly, the proteins also have similar biochemical properties. Like tubulin, FtsZ assembles in vitro in a head to tail fashion to form single stranded protofilaments, which can further assemble into bundles, sheets or rings. The protofilaments are also highly dynamic and go through cycles of turnover/polymerization, regulated by the binding and hydrolysis of GTP. See Chapter 10.1007/978-3-319-53047-5_5 for a detailed description of FtsZ polymerization dynamics.

### FtsZ Visualization During Growth and Sporulation of *B. subtilis*

The ability of FtsZ to form tubulin-like protofilaments and protofilament bundles raised important questions about the abundance, assembly and dynamics of the protein in vivo. Estimations of protein abundance have suggested about 2000–6000 molecules per cell in *B. subtilis* (Feucht et al. 2001; Ishikawa et al. 2006; Muntel et al. 2014), giving a protein concentration of about 2–6 μM, well above the in vitro critical concentration for assembly (e.g. 0.72 μM for *E. coli* FtsZ; Chen et al. 2012). Also, given a protofilament subunit repeat length of 4.3 nm (Oliva et al. 2004), there is enough FtsZ to circumnavigate the cell about 3–10 times.

Several labs have investigated the localization of FtsZ in *B. subtilis*. Wang and Lutkenhaus (1993) used immunogold electron microscopy to demonstrate association of FtsZ with the leading edge of the invaginating cell division structure. The immunofluorescence studies of (Levin and Losick 1996) confirmed the presence of FtsZ bands (presumed to be rings) at the expected position near mid cell in vegetative cells but also unexpectedly, near both poles of early sporulating cells. This turns out to be a key feature of the mechanism used by *B. subtilis* to achieve asymmetric cell division during sporulation, which will be discussed in detail below. Several reports have highlighted the possible role of helical FtsZ structures as intermediates in assembly or constriction at cell division sites (Ben-Yehuda and Losick 2002; Feucht and Errington 2005; Peters et al. 2007; Strauss et al. 2012). Helical FtsZ structures are most prominent during the transition from vegetative growth to sporulation in *B. subtilis*, during which the site of division shifts from mid cell to near the pole. The mid cell Z ring appears to transform into a helix which grows length-wise, before breaking down into two short helices, one near each pole. Each helix then coalesces into a ring (Ben-Yehuda and Losick 2002). Therefore, each sporulating cell assembles two Z rings, one at each pole, but only one develops into a septum (see below). Several mutations in *ftsZ* have been described that promote a tendency to form spiral Z rings and similarly shaped division events (Feucht and Errington 2005; Michie et al. 2006), suggesting that the helical configuration has functional relevance. Peters et al. (2007) also described helical configurations in vegetative cells, based on modified immunofluorescence imaging methods. On the other hand, several higher resolution imaging methods, including super-resolution fluorescence imaging and cryo-EM of *B. subtilis* and other organisms have suggested that FtsZ rings may be more complex, and beaded or discontinuous (Jennings et al. 2011; Strauss et al. 2012; Li et al. 2007; Min et al. 2014).

Dynamic movement of FtsZ rings has been observed by time-lapse imaging (Strauss et al. 2012) but more quantitative and perhaps surprising information came from fluorescence recovery after photobleaching (FRAP) experiments. Erickson and colleagues established that FtsZ subunits in Z rings, either pre-constriction or during constriction, turn over with a half time of about 8 seconds (Anderson et al. 2004). This emphasises the likely importance of dynamics in FtsZ function but also, the difficulty in imaging such structures with the need for both high spatial and temporal resolution. Löwe’s lab have recently described compelling evidence for the formation of regular circumferential bands of FtsZ in which the protofilaments are connected by regular lateral contacts for 2 Gram negative bacteria, *E. coli* and *Caulobacter crescentus*, as well as in an in vitro system. These observations support a model for constriction involving filament sliding (Szwedziak et al. 2014). It remains to be seen whether this model can be extended to *B. subtilis* and other Gram positive bacteria, but it seems unlikely that the fundamental features of FtsZ function in bacterial division are not well conserved.

### The *B. subtilis* Divisome

The *B. subtilis* divisome has been studied in considerable detail: some properties of the proteins thought to contribute directly or indirectly to divisome function in this organism are described in Table 3.2. These proteins have been identified through homology to known division proteins in other organisms, by biochemical pull downs or through various genetic screens.

**Table 3.2:** Proteins of the *B. subtilis* divisome and its regulators

Protein	MW (kDa)	Location^a^	Comments	Key references
FtsZ	40	C	Tubulin-like protein. Assembles into protofilaments and higher order structures to generate the “Z ring” at the division site. Recruits other divisome proteins to the ring.	Beall et al. (1988), Beall and Lutkenhaus (1991), and Wang and Lutkenhaus (1993)
FtsA	48	C	Actin / HSP70 superfamily ATPase. Dimerises and can form higher order structures. C-terminal amphipathic helix promotes membrane association. Direct interaction with FtsZ, which contributes to membrane association of the Z ring.	Beall and Lutkenhaus (1991), Feucht et al. (2001), Jensen et al. (2005) and Ishikawa et al. (2006)
SepF	17	C	Forms regular 50 nm diameter rings in vitro and interacts directly with FtsZ in vitro, promoting FtsZ bundling. Membrane targeting domain contributes to membrane association of the Z ring.	Hamoen et al. (2006) and Gündoğdu et al. (2011)
ZapA	9.0	C	Widely conserved protein that promotes Z ring formation by direct interaction with FtsZ.	Gueiros-Filho and Losick (2002)
EzrA	65	C	N-terminal transmembrane anchor. Cytosolic domain has a spectrin-like fold. Interacts with FtsZ, contributing to membrane association of the Z ring. Additional role in cell elongation via interactions with PBP 2B and GpsB.	Levin et al. (1999), Haeusser et al. (2004), Claessen et al. (2008), and Cleverley et al. (2014)
GpsB	11	C	DivIVA-related protein involved in both cell elongation and cell division. Interacts with the major PG synthase, PBP 1, and thought to be involved in shuttling of this protein between elongation and division complexes. Synthetic lethal in combination with *ftsA* mutation. Synthetic “sick” in combination with *ezrA*. EzrA-SepF interaction probably important for shuttling.	Claessen et al. (2008) and Tavares et al. (2008)
FtsL	13	E	Bitopic membrane protein with short extracytoplasmic coiled-coil-like domain. Target of several cell division regulatory mechanisms. Unstable protein subject to degradation by a regulated intramembrane proteolysis (RIP) process involving YluC protease. Stability also regulated by interactions with DivIC and DivIB.	Daniel et al. (1998), Daniel and Errington (2000), Sievers and Errington (2000a, b), Kawai and Ogasawara (2006), Bramkamp et al. (2006) and Daniel et al. (2006)
DivIB	30	E	Bitopic membrane protein with large extracellular domain. Structural data from other organisms suggests two domains, one of which resembles the POTRA domain often involved in protein protein interactions. Complex pattern of interactions with FtsL and DivIC. Homologue called FtsQ in *E. coli*.	Beall and Lutkenhaus (1989), Harry and Wake (1989, 1997), Katis and Wake (1999), Katis et al. (2000), Daniel and Errington (2000) and Daniel et al. (2006)
DivIC	15	E	Bitopic membrane protein with short extracytoplasmic coiled-coil-like domain. Interacts with FtsL and DivIB. Likely homologue confusingly called FtsB in *E. coli*.	Katis et al. (1997), Katis and Wake (1999), Katis et al. (2000), Sievers and Errington (2000b), Robson et al. (2002) and Daniel and Errington (2000)
FtsW	44	I	Integral membrane protein closely related to RodA involved in cell elongation.	Lu et al. (2007)
Pbp2B	79	E	Penicillin binding protein. Monofunctional (class B) transpeptidase specifically required for cell division.	Yanouri et al. (1993), Daniel et al. (1996) and Daniel and Errington (2000)
DivIVA	19	C	Coiled coil protein with weak similarity to eukaryotic tropomyosins. Targeted to division sites and cell poles at least in part by sensing membrane curvature. Membrane interaction through conserved N-terminal domain containing essential tryptophan residue. Involved in a range of cell pole associated functions in Gram positive bacteria.	Cha and Stewart (1997), Edwards and Errington (1997), Hamoen and Errington (2003) and Lenarcic et al. (2009), Ramamurthi and Losick (2009) and Van Baarle et al. (2013)
MinC	25	C	Widely conserved division inhibitor acting on FtsZ and possibly other steps in division.	Reeve et al. (1973), Levin et al. (1992), Marston and Errington (1999) and Gregory et al. (2008)
MinD	29	C	Widely conserved indirect division inhibitor that works by spatial regulation of MinC protein. Poorly characterised additional role in chromosome segregation during sporulation.	Reeve et al. (1973), Levin et al. (1992), Marston et al. (1998) and Marston and Errington (1999), Kloosterman et al. (2016)
MinJ	44	I / C	PDZ-domain protein targeted to cell poles by interaction with DivIVA (at least). Required for correct spatial localization of the MinCD complex and thus the regulation of cell division.	Patrick and Kearns (2008), Bramkamp et al. (2008) and Van Baarle and Bramkamp (2010)
Noc	33	C	Site-specific DNA binding protein. Inhibitor of division. Major factor effecting nucleoid occlusion.	Wu and Errington (2004), Wu et al. (2009) and Adams et al. (2015)
WhiA	36	C	Enigmatic nucleoid associated factor. *whiA* mutation causes severe filamentation when combined with *zapA*, *ezrA* or various regulatory proteins of cell division.	Surdova et al. (2013)
SpoIIE	92	C/I	Bifunctional sporulation-specific protein. C-terminal kinase domain regulates prespore-specific gene expression. C-terminal domain required for efficient switch in cell division position from mid cell to sub-polar position, probably via a direct interaction with FtsZ.	Arigoni et al. (1995), Feucht et al. (1996), Wu et al. (1998), Lucet et al. (2000), Carniol et al. (2005) and Bradshaw and Losick (2015)
MciZ	4.0	C	Mother cell-specific inhibitor of FtsZ assembly. Caps FtsZ protofilaments at the “minus” end.	Handler et al. (2008) and Bisson-Filho et al. (2015)
RefZ	24	C	Site-specific DNA-binding protein that contributes to precise relative positioning of chromosome and asymmetric division site during sporulation.	Wagner-Herman et al. (2012) and Miller et al. (2015)

^a^ *C* cytosolic, *I* integral membrane, *E* extracytoplasmic

Imaging experiments suggest that the divisome assembles in at least two distinct steps (Gamba et al. 2009). In the first step, which seems to involve mainly cytosolic factors, a “ring” of FtsZ protein assembles, in parallel with the recruitment of “early” divisome proteins FtsA, SepF, ZapA and EzrA. After a delay representing about 20% of the cell cycle, the second step of assembly takes place, in which the “late” proteins are recruited. These are mainly proteins with major extracellular domains or integral membrane proteins. Various regulatory proteins, including GpsB, DivIVA, MinJ, MinD and MinC arrive at about the same time or slightly later, possibly being dependent on initiation of membrane or PG ingrowth. Ishikawa et al. (2006) detected interactions between the various early proteins in a series of biochemical pull-down experiments.

Three “early” cytosolic proteins appear to promote the formation of a functional Z ring in *B. subtilis* – FtsA, SepF and ZapA. FtsA was identified by its conserved location immediately upstream of and adjacent to FtsZ (Beall et al. 1988). Unlike *E. coli*, *ftsA* mutants of *B. subtilis* are viable, though they are substantially deficient in division (Beall and Lutkenhaus 1992). FtsZ still localizes at regular intervals but most of the Z rings are abnormal, often appearing as multiple diffuse bands rather than one clear, strong band (Jensen et al. 2005). Purified *B. subtilis* FtsA binds and hydrolyses ATP (Feucht et al. 2001) but little more work has been done on this protein so far. Using the *Thermotoga maritima* protein, Löwe and colleagues have demonstrated that even though FtsA has a different subdomain architecture to actin, the protein can form canonical actin-like protofilaments in vitro (Van Den Ent and Löwe 2000; Szwedziak et al. 2012). FtsA interacts specifically with the C-terminal domain of FtsZ. Despite a great deal of work over nearly 2 decades, little is known about the precise function of *ftsA* other than that it can form high MW dynamic complexes of various kinds with FtsZ (e.g., Loose and Mitchison 2014). Perhaps its best defined function lies in membrane association, which occurs through a C-terminal amphipathic helix (Pichoff and Lutkenhaus 2005) and enables the protein to anchor the Z ring to the membrane (Szwedziak et al. 2014). Interestingly, this interaction with the membrane is strongly dependent on the membrane potential (Strahl and Hamoen 2010).

Genetic and biochemical experiments suggest that SepF protein provides a second membrane anchor for the Z ring in Gram positive bacteria. *sepF* was discovered simultaneously in two labs by different methods. Ishikawa et al. (2006) identified the SepF protein in pull-down experiments using FtsZ, FtsA, EzrA and ZapA as bait. Yeast 2-hybrid experiments detected the formation of a SepF-SepF self interaction, as well as an interaction with FtsZ. Meanwhile, Hamoen et al. (2006) identified *sepF* as a candidate cell division gene from its conserved position (in Gram positive bacteria) between *ftsZ* and *divIVA*. Deletion of the gene gives a mild reduction in division frequency but the division septa formed are thick and morphologically abnormal. Mutation of *sepF* turned out to be lethal in the presence of mutations in *ftsA* or another division associated gene, *ezrA* (Ishikawa et al. 2006; Hamoen et al. 2006). In vitro, SepF protein assembles into large and regular protein rings with a diameter of about 50 nm: these rings are able to bundle FtsZ protofilaments into long tubular structures (Gündoğdu et al. 2011). Detailed structural analysis of the protein (Duman et al. 2013) suggests that the N-terminal region, like FtsA, contains a membrane associating amphipathic helix, whereas the C-terminal domain is globular and responsible for both the formation of SepF rings and association with FtsZ. Duman et al. (2013) suggest that the amphipathic helices of FtsA and SepF both serve to promote association of the Z ring with the leading edge of the septum, since this region contains positively curved (convex) membrane into which the helices can readily insert. Duman et al. (2013) proposed a model in which SepF polymers bind as arc onto the convex leading edge of the nascent division septum and maintain the Z ring in this position by bundling FtsZ protofilaments. However, this model is slightly unsatisfactory in leaving open the question of why SepF makes complete rings in vitro.

ZapA is a low MW (9 kDa) positive regulator of FtsZ assembly. It was identified in a screen for genes which, when overexpressed, could overcome the cell division block caused by overproduction of MinD (Gueiros-Filho and Losick 2002). Absence of ZapA gives no discernible phenotype under normal conditions but causes a severe division block in cells producing lower than normal levels of FtsZ, or lacking the *ezrA*, *divIVA* or *whiA* genes (Gueiros-Filho and Losick 2002; Surdova et al. 2013). ZapA interacts directly with FtsZ and, in vitro, it promotes FtsZ polymerisation as well as lateral association, yielding both single and bundled filaments (Gueiros-Filho and Losick 2002; Low et al. 2004). A temperature-sensitive mutant of FtsZ (FtsZ(Ts1)), defective in lateral association between FtsZ protofilaments at high temperatures, can be rescued by overexpressing ZapA. This supports the proposed function of ZapA as a promoter of FtsZ bunding (Monahan et al. 2009). ZapA of *Pseudomonas aeruginosa* forms dimers or tetramers in solution but oligomerizes at high concentrations; a property that could support its function as an effective FtsZ cross-linker (Low et al. 2004).

The *ezrA* gene, which is present only in Gram positive bacteria, was identified by mutations suppressing the division phenotype of a thermosensitive *ftsZ* allele (Levin et al. 1999). *ezrA* mutants can tolerate reduced levels of active FtsZ and the gene name derives from the observation that the *ezrA* single mutant makes extra Z-rings. The protein has an unusual topology with a single N-terminal transmembrane span followed by a major domain that is cytosolic. Curiously, one protein that shares this unusual topology is *E. coli* ZipA, which is an essential FtsZ-interacting protein in *E. coli* (Hale and De Boer 1997), but it seems that EzrA and ZipA are otherwise unrelated in sequence. *ezrA* mutants also have a slightly reduced cell diameter, indicating a mild defect in cell elongation. Genetic experiments suggest that this may be due to incorrect regulation of the activity of the major PBP (PBP 1) involved in synthesis of PG during both elongation and division (Claessen et al. 2008). The results of detailed mutational analysis of the gene suggest that different regions of the large protein contribute in different ways to the regulation of Z ring dynamics (Haeusser et al. 2007; Land et al. 2014). The crystal structure of the cytoplasmic domain of EzrA was recently solved (Cleverley et al. 2014) and shown to be similar to that of eukaryotic spectrins, comprising multiple, connected repeats of antiparallel α-helices, forming a complete semi-circle of 12 nm diameter. Spectrins are cytoskeletal proteins that can form two-dimensional polygonal networks lining the membrane, and they help maintain plasma membrane integrity and cytoskeletal structure in eukaryotic cells. The formation of a semi-circle could enable both the C-terminal four-helix bundle and the N-terminal transmembrane domain to interact with the membrane at the same time. Structural modelling indicates that an antiparallel dimer of EzrA molecules, as found in some crystal structure forms, could trap a paired FtsA-FtsZ protofilament inside the arch. In principle, this could serve to both anchor the protofilaments to the membrane and or locally prevent the formation of protofilament bundles.

Because FtsZ protein is thought to be indirectly associated with the cell membrane, through its interactions with FtsA, SepF and possible EzrA, it seems likely that most of the remaining divisome proteins, which are largely integral membrane or extracytoplasmic proteins (summarised in Table 3.2), do not interact directly with FtsZ. Their functions are probably concerned mainly with membrane dynamics, or peptidoglycan synthesis and turnover and will not be discussed in detail here.

### Regulation of Z Ring Formation and Cell Division

Cell division needs to be tightly coordinated with other cell cycle events, particularly chromosome replication and segregation. Recently, the field of bacterial cell cycle regulation has been invigorated by the unexpected discovery that cell size homeostasis is achieved by an “adder” process in which new born cells grow by a relatively fixed length increment before dividing again, rather than by measuring a “division mass”, according to a decades old dogma (Campos et al. 2014; taheri-araghi et al. 2015). The key questions now concern how the length increment is measured by the cell and used to regulate divisome function. In *B. subtilis* the intracellular concentration of FtsZ stays constant throughout the cell cycle and, although the frequency of Z ring formation varies with growth rate, the levels of FtsZ are unaffected. Artificially increasing the level of FtsZ in *B. subtilis* cells only leads to a small increase in Z ring frequency (Weart and Levin 2003).

One factor that could be involved in buffering the levels of available FtsZ is the two-component ATP-dependent protease, ClpXP. ClpX is a member of the AAA+ (ATPases associated with various cellular activities) family of ATPases. It recognizes and unfolds specific protein substrates and transfers the unfolded protein to the serine protease ClpP for degradation (Sauer et al. 2004). ClpXP is thought to participate in the regulation of FtsZ assembly by maintaining the pool of subunits available for ring formation (Weart et al. 2005; Camberg et al. 2009; Dziedzic et al. 2010). In *B. subtilis* ClpX inhibits FtsZ polymerization *in vivo* and *in vitro* in an ATP-and ClpP-independent manner but does not degrade it, though ATP hydrolysis has been shown to be required for maximum inhibition (Weart et al. 2005; Haeusser et al. 2009). This is in contrast to *E. coli* in which ClpX modulates the level of FtsZ by degrading FtsZ (Camberg et al. 2009).

Several spatial and or temporal regulators of divisome function have been identified, mainly acting by negative regulatory mechanisms.

### Nucleoid Occlusion (NO)

The fact that cell division tends not ever to bisect the nucleoid, even in cells with perturbations in chromosome replication or organization, led Woldringh and colleagues to postulate a negative regulation exerted by the nucleoid, potentially by the DNA itself (Mulder and Woldringh 1989; Woldringh et al. 1991), which Rothfield later termed “nucleoid occlusion” (Cook et al. 1989). About 10 years ago, protein factors contributing to NO were identified almost simultaneously in *B. subtilis* (*noc*) and *E. coli* (*slmA*) (Wu and Errington 2004; Bernhardt and De Boer 2005): surprisingly, they were unrelated proteins that turned out to have different modes of division inhibition. *B. subtilis noc* was identified serendipitously as a factor that had a synthetic lethal division phenotype when combined with mutations affecting the Min system (which is described below). Three lines of evidence suggested that Noc protein was a NO factor: first, the protein had a classical helix-turn-helix motif and bound tightly to DNA; second, overexpression of Noc inhibited division; third, *noc* mutants had an increased frequency of nucleoid “guillotining” when DNA replication or segregation was perturbed (Wu and Errington 2004). Later work established that Noc is a site-specific DNA-binding protein with recognition sites (Noc binding sites; NBS) distributed all over the chromosome, except in the replication terminus region, where binding sites are scarce (Wu and Errington 2004; Wu et al. 2009). Noc appears to have been derived by gene duplication and divergence from the ParB (Spo0J) protein in Firmicutes, and like Spo0J it spreads from its primary binding sites to form arrays which are important for its function. While the N-terminal domain of Spo0J (ParB) is involved in interaction with its partner protein Soj, the N-terminal domain of Noc contains an amphipathic helix, which enables it to associate with the cell membrane (Adams et al. 2015). Both overexpression and deletion of Noc affect division at the level of FtsZ assembly (Wu and Errington 2004). It seems that the formation of Noc arrays around NBSs enhances membrane association and that recruitment of these DNA-Noc arrays to the membrane prevents the local formation of FtsZ ring assemblies (Adams et al. 2015). It is possible that Noc works by enhancing a natural NO system, akin to that originally described by Woldringh, in which the presence of chromosomal DNA excludes accumulation or formation of high MW divisome complexes.

### The Min System

NO prevents division from occurring in the vicinity of the nucleoid, but it cannot protect the nucleoid free regions at the nascent and old cell poles. The system that prevents polar division is called Min and was first discovered *via E. coli* mutants that frequently divide near to the cell pole, giving small anucleate cells called minicells (Adler et al. 1967). The *B. subtilis* MinC and MinD proteins were identified by sequence homology to their *E. coli* counterparts (Levin et al. 1992). The *B. subtilis* Min system is now known to consist of at least four proteins: MinC, MinD, MinJ and DivIVA. MinC is an FtsZ inhibitor which interacts directly with FtsZ. In vitro studies have shown that like *E. coli* MinC, the *B. subtilis* protein inhibits FtsZ bundle formation by disrupting lateral interactions between protofilaments (Dajkovic et al. 2008; Scheffers 2008; Blasios et al. 2013), though *E. coli* MinC has been shown to also destabilize FtsZ protofilaments (Hu et al. 1999; Shen and Lutkenhaus 2010). MinD is a membrane-associated activator of MinC. Both MinC and MinD are relatively well conserved among bacteria. The two proteins form a heterotetrameric complex and in *B. subtilis* are recruited to the division site and the cell poles by MinJ, which in turn associates with the “topological specificity” determinant DivIVA (Edwards and Errington 1997; Marston et al. 1998; Bramkamp et al. 2008; Patrick and Kearns 2008). *E. coli* lacks counterparts of the MinJ and DivIVA proteins and instead uses an amazing oscillating MinCD mechanism to prevent division at the cell poles (Lutkenhaus 2007).

The key feature of DivIVA that enables it to spatially control the Min inhibitory effect lies in its targeted localization to division sites and cell poles. DivIVA oligomers have affinity for high negative membrane curvature, which normally occurs only at invaginating division septa or recently completed cell poles (Lenarcic et al. 2009; Ramamurthi and Losick 2009; Eswaramoorthy et al. 2011). It is probably recruited to the site of division as soon as membrane invagination begins, due to divisome constriction. Therefore, accumulation of DivIVA at the division site is dependent on the presence of a functional divisome but once curvature has been generated, the rings of DivIVA, one on each side of the growing septum, are no longer affected by contraction of the divisome (Eswaramoorthy et al. 2011). Upon completion of septation, the divisome disassembles and the septum splits to generate new cell poles for the two daughter cells. In cells that are not dividing, DivIVA-GFP is concentrated at the hemispherical cell poles (Eswaramoorthy et al. 2011) but in dividing cells, DivIVA is remodelled and a portion of the DivIVA molecules remain at the pole, while some protein migrates to the new division site (Eswaramoorthy et al. 2011; Bach et al. 2014). The main structural feature of DivIVA is a parallel coiled coil, similar to the yeast tropomyosin Cdc8, a eukaryotic cytoskeletal protein involved in cytokinesis (Edwards et al. 2000; Oliva et al. 2010). This major C-terminal portion of DivIVA resembles the crescent shape of eukaryotic BAR domains normally found at the interface between the actin cytoskeleton and lipid membranes, which bind to curved membranes and also introduce curvature (Oliva et al. 2010). This raises the possibility that DivIVA senses membrane curvature using a mechanism similar to the Bar domain proteins. Structural and genetic evidence suggest that membrane interaction occurs via a hairpin structure with conserved exposed basic and hydrophobic residues in the N-terminal domain of the protein (Oliva et al. 2010).

MinJ is presently the least well characterised component of the Min system. It has 6 transmembrane helices with both N- and C-termini in the cytoplasm. The C-terminal globular portion of the protein comprises a classical PDZ domain; a protein fold often involved in protein-protein interactions (Van Baarle and Bramkamp 2010). MinJ can interact with both DivIVA and MinD, based on 2-hybrid experiments (Patrick and Kearns 2008; Bramkamp et al. 2008), suggesting that MinJ is the immediate polar target for recruitment of MinD, rather than DivIVA (Bramkamp et al. 2008).

As originally described by Marston et al. (1998) and reinforced by subsequent papers (Gregory et al. 2008; Bramkamp et al. 2008; Van Baarle and Bramkamp 2010), DivIVA and presumably now MinJ are recruited to mid cell soon after the initiation of division. MinJ, in turn, recruits the MinCD complex, which has no effect on the ongoing division but is poised to disassemble the divisome as division is completed, and or prevent the assembly of a new division complex. Although in general it appears that little, if any, of these proteins are retained at completed “old” cell poles, some activity is probably retained to prevent inappropriate minicell divisions from occurring there.

The mechanism of action of the MinCD inhibitor is not yet fully understood, despite over 20 years of study. MinD is a member of the Walker A cytoskeletal ATPase (WACA) family, a group of cytoskeletal proteins thought to be unique to bacteria (Löwe and Amos 2009; Shih and Rothfield 2006; Michie et al. 2006; Pilhofer and Jensen 2013). Characteristic of this family is a ‘deviant’ Walker A motif – KGGXXGKT’ containing two conserved lysines, both important for binding and hydrolysis of ATP (Lutkenhaus 2012). As in *E. coli*, the ATPase activity of *B. subtilis* MinD is required for membrane binding and activation of MinC (Karoui and Errington 2001), but biochemical details of how the inhibitory activity of the MinCD complex is regulated remain elusive. One interesting recent development has been the report that *E. coli* MinC and MinD form alternating copolymeric cytomotive filaments with structural similarity to septins (Ghosal et al. 2014). Septins are a group of eukaryotic GTP-binding cytoskeletal proteins that polymerize into hetero-oligomeric protein complexes and play many important roles, including serving as membrane scaffolds for protein recruitment and as diffusion barriers for subcellular compartmentalization (Mostowy and Cossart 2012). It is not clear whether the *B. subtilis* homologues behave similarly or what the functional significance of the copolymer organization is.

### Nutritional Regulation of Cell Division

In *B. subtilis* and probably many other bacteria, cell size is regulated according to the growth rate, such that fast growing cells are, on average, larger than slow growing cells (Sharpe et al. 1998). Weart et al. (2007) identified the UgtP protein as a key metabolic regulator of cell division in *B. subtilis*. UgtP is responsible for synthesis of glucolipids using UDP-glucose as a substrate. Mutations in the *ugtP* gene, or genes upstream in the UDP glucose synthetic pathway (*pgsC* or *gtaB*) had a small cell phenotype, in which both FtsZ ring formation and cell division occur at a smaller average cell size than in the wild type. UgtP turned out to interact directly with FtsZ in vitro and in vivo, and its inhibitory effect on FtsZ assembly is stimulated by UDP-Glucose (Weart et al. 2007). Under nutrient rich conditions UgtP levels are increased, as is the availability of its UDP-Glucose substrate, leading to an inhibition/delay in assembly of the FtsZ ring.

Monahan et al. (2014) identified a similar but quite distinct regulatory effect on cell division involving central carbon metabolism. They showed that a temperature sensitive *ftsZ* mutant could be rescued by mutations in genes encoding pyruvate kinase (*pyk*) or phosphoglycerate kinase (*pgk*) and established that these mutations work by limiting the supply of pyruvate from glycolysis. They identified the E1α subunit of pyruvate dehydrogenase, which uses pyruvate as a substrate in generating acetyl-CoA. Localization of E1α was found to shift between nucleoid associated and nucleoid excluded depending on the availability of nutrients (high vs low, respectively). Various genetic tests were consistent with a model in which E1α is a positive regulator of FtsZ ring formation helping to couple this to sensing of nutrient availability. Molecular details of the putative interaction between the various players in this process remain to be worked out.

### Z Rings and Cell Division During Sporulation

Early in sporulation the cell division cycle is substantially modified to pave the way for generation of the distinct prespore and mother cell progeny and their subsequent differentiation. As mentioned above, this involves a repositioning of FtsZ rings away from the normal mid cell position to the cell poles (Errington 2003). The following section focuses mainly on events involving FtsZ. The key cell cycle changes, which are controlled by global changes in transcription in response to starvation, are as follows. First, medial division is blocked by a mechanism that is presently unclear, but a major change in chromosome configuration – the formation of a structure called the axial filament (Ryter 1965; Bylund et al. 1993) – probably contributes, through a nucleoid occlusion effect. Instead FtsZ rings are directed to sub-polar positions at each end of the cell (Levin and Losick 1996). Formation of these polar Z-rings requires both a small upregulation of FtsZ synthesis and the synthesis of a sporulation-specific protein SpoIIE (Ben-Yehuda and Losick 2002; Feucht et al. 1996) (see below). The upregulation of *ftsZ* occurs via a promoter controlled by the σ^H^ form of RNA polymerase, which is active only in stationary phase and sporulation (Gholamhoseinian et al. 1992; Gonzy-Tréboul et al. 1992).

Mutations in several key regulatory genes of sporulation give rise to an interesting phenotype called “disporic”, in which prespore-like cells form at both poles of the cell (Piggot and Coote 1976). Lewis et al. (1994) showed that in these cells the asymmetric division events occurred sequentially, with the first preceding the second by about 20 min. This suggested that the Z rings at the two poles develop at different rates, ultimately contributing to the generation of asymmetry – whichever potential divisomes matures first defines the pole which the prespore cell forms (Lewis et al. 1994). The polar sporulation septum differs from a normal vegetative septum in having a much thinner layer of PG (Ryter 1965; Illing and Errington 1991; Tocheva et al. 2013). This thinning may be related to the fact that a little while later, the PG needs to be hydrolysed to enable the remarkable process of prespore engulfment to occur: the small prespore is engulfed by the mother cell to produce a cell within a cell, similar to eukaryotic phagocytosis (Illing and Errington 1991; Tocheva et al. 2013). In addition to FtsZ (Beall and Lutkenhaus 1991), FtsA is probably also required for sporulation division (Beall and Lutkenhaus 1992), though curiously, the latter protein only appears to accumulate at one of the polar potential division sites – presumably the one that goes on to support division (Feucht et al. 2001). DivIB, DivIC and FtsL, at least, are also required for formation of the sporulation septum (Levin and Losick 1994; Daniel et al. 1998; Feucht et al. 1999) but whether the other vegetative divisome proteins are also required for the sporulation septum has not been systematically studied. How the polar septum is formed despite continued presence of the Noc, MinCDJ and DivIVA proteins is also not clear.

The large (92 kDa) SpoIIE protein plays two distinct critical roles in the prespore developmental programme. In addition to its role in asymmetric septation, it is also essential for activation of the first compartment-specific transcription factor, σ^F^, in the prespore (Duncan et al. 1995; Arigoni et al. 1996; Feucht et al. 1996). The N-terminal domain of SpoIIE contains 10 predicted transmembrane spans. This is followed by a central regulatory domain and a C-terminal PP2C phosphatase domain. SpoIIE is recruited to both polar Z rings sequentially (Arigoni et al. 1995; Levin et al. 1997; Wu et al. 1998), probably via a direct interaction with FtsZ (Lucet et al. 2000). The precise role of SpoIIE in FtsZ assembly is still not clear. Absence of SpoIIE causes a delayed and reduced frequency of polar divisions, as well as a vegetative-like thickening of the septa that are formed (Illing and Errington 1991; Barák and Youngman 1996, Feucht et al. 1996; Khvorova et al. 1998; Ben-Yehuda and Losick 2002; Carniol et al. 2005). Crucially, after septation, the SpoIIE protein from the septum is sequestered into the smaller prespore compartment (Wu et al. 1998; Campo et al. 2008; Bradshaw and Losick 2015), where the phosphatase domain helps to drive the activation of σ^F^ specifically in that compartment. Regulation of SpoIIE is highly complex and involves association with and release from the divisome, recruitment at the adjacent cell pole by interaction with DivIVA, oligomerization and controlled proteolysis (Bradshaw and Losick 2015).

At the transcriptional level, formation of a polar septum, through the localized action of SpoIIE phosphatase, triggers the prespore localized activation of σ^F^, which then turns on the early prespore programme of gene expression. One of the newly expressed genes, *spoIIR*, encodes a factor that triggers the activation of a different sigma factor, σ^E^, in the mother cell compartment. Among the proteins made as a result of transcription by σ^E^-RNA polymerase is a specific inhibitor of *ftsZ* assembly, called MciZ (Handler et al. 2008) that appears to work as a protofilament capping protein (Bisson-Filho et al. 2015). MciZ helps to block the utilization of the second polar FtsZ ring located in the mother cell compartment. Three other σ^E^-dependent proteins (SpoIID, SpoIIM and SpoIIP) also facilitate the formation of a second polar division, but apparently by working downstream on PG synthesis (Eichenberger et al. 2001).

### FtsZ Inhibitors as Potential Antibiotics

FtsZ of *B. subtilis* and closely related Gram positive bacteria, including *Staphylococcus aureus*, is susceptible to inhibition by a family of related benzamide compounds, with potential for use as antibiotics. These compounds bind to an allosteric site in the protein and apparently trap the protein in the “open” state, which promotes protofilament assembly (Haydon et al. 2008; Tan et al. 2012). In vivo, this results in the formation of multiple discrete foci of FtsZ, which recruit all tested downstream divisome proteins (4 early and 4 late) (Haydon et al. 2008; Adams et al. 2011). However, productive Z rings are not formed, leading to a complete division block. The potential clinical use of these compounds has not yet been evaluated.

### L-Form (Cell Wall Deficient) Bacteria

Despite the extraordinary complexity of the wall, its various important functions, and its role as the target for many powerful antibiotics, it is surprisingly easy for *B. subtilis* to lose its wall. Only one or two mutations are needed to enable *B. subtilis* (and many other organisms; Mercier et al. (2014)) to switch into a wall deficient mode called L-form (Leaver et al. 2009; Mercier et al. 2013; Kawai et al. 2015). L-forms require an osmoprotective medium to prevent them from incurring osmotic lysis and they have pleomorphic shapes, due to lack of the rigid cell wall. Remarkably, L-forms can tolerate the complete deletion of many genes that are normally essential for growth and division, including both FtsZ and the complete set of MreB homologues (Leaver et al. 2009; Mercier et al. 2012). They divide by a blebbing mechanism that requires only an increase in membrane synthesis (Mercier et al. 2013). Apart from interest in L-forms as potential biotechnological devices or possibly as agents responsible for certain infectious diseases, they are likely to be useful in basic studies of cell wall elongation and division through their ability to tolerate the disruption of many genes that are normally essential (Kawai et al. 2014).

### The Future

Some of the open questions about the FtsZ system are similar to those of MreB. The advent of cryo-EM tomography is beginning to resolve the nature of the FtsZ ring (Szwedziak et al. 2014) but resolution of the detailed structure in vivo remains probably the biggest impediment to understand divisome function. Although various temporal and spatial regulators are now known and have been subjected to detailed study, again, understanding of their precise mechanism of action will probably await resolution of the Z-ring structure problem. Furthermore, even in the absence of the key negative regulators (MinCD and Noc), residual cell divisions still tend to occur between replicated chromosomes (Wu and Errington 2004; Rodrigues and Harry 2012), indicating the existence of as yet unidentified regulatory factors.

Once the structure of the Z ring has been resolved many details of the division process will need to be worked out, including the enigmatic roles of several membrane associated divisome proteins, such as DivIB, DivIC, FtsL and FtsW. Finally, it will be interesting to resolve how the division machinery is modified in order to bring about the various subtle changes associated with the asymmetric division of sporulating cells. How is mid cell division blocked? How is polar division promoted: in particular, how are the normal activities of the NO and Min systems overridden? Finally, how does the cell regulate the differential thickness of the vegetative vs sporulation septa?
